# Homœopathic Cures

**Published:** 1891-09

**Authors:** 

**Affiliations:** Berlin


### HOMŒOPATHIC CURES.

### Dr. Dahlke, Berlin.

(*Zeitschrift der Berliner Vereins Hom. Aerzte, Vol. X*, 3.)

1. A woman suffered for several weeks from severe tearing pains in the face. Home treatment failed, and the extraction of several teeth gave no relief. The pains were exclusively on the left side and radiated toward the ear, relieved by heat; worse from very cold draught, by cold or warm food; sleepless nights. Rhus-tox. has amelioration by heat, worse at night and by cold air. Colocynth more than Rhus favors the left side and is considered a sovereign remedy in prosopalgia.* Sensitiveness to draughts may hint at China, which also has the sensitiveness of the scalp and of the roots of the hair, for the woman was afraid to comb her hair. Her anemia hinted at Pulsatilla, but its pain, though tearing, is characteristic, a sensation as if the nerve were drawn tense and then suddenly relaxed, and Puls. had amelioration in fresh air. She remarked incidentally that the pains are aggravated by washing her hands in cold water, and this remark decided the selection for Rhus$^{5x}$, three drops every hour, which quickly gave relief. How often do we fail in our examinations, because the patient considers of no account a symptom which is really the key-note of the case, and in an era where suggestion plays a decided part, one ought to be very careful not to suggest symptoms to the mind of the patient.

2. Early one morning Dahlke was called to a gentleman suffering from cardialgia. On account of relief from bending backward Belladonna was given. After four hours no better; Phosphor.$^{5x}$, with some amelioration, but during the day pains

---

* So are Spigelia left side, Kalmia right side, and Cidron both sides, but more periodicity. Though one is wrong to have favorites, still favorable experiences render one lenient to such abuse, and I plead guilty. Colocynth has amelioration by strong pressure, but aggravation after its removal, aggravation by motion, while Rhus patient cannot keep quiet. In these unilateral neuralgias Gelsemium (200) will often nip the whole disease in the bud, and is too much neglected.—S. L.

returned, but not so severe. On the fourth day fullness in the gastric region, pressing pain, eructations without relief, constant heat in his ears. As this symptom is found in China$^{3}$, a few drops every hour was given. A few months afterward he returned to have the vial refilled, as the remedy worked like magic. Mere symptom covering and mere accident ! some would say, and it is hardly possible that China is the only drug which causes red and hot ears, for one meets it in Sanguinaria with its vasomotory extravaganzas, in Lycopodium, Magnesia, and Camphor, and Ficus-indica has hot ears. Peculiar characteristic symptoms of a case are only of value when in full concordance with the other symptoms, and the more we hold of them in our memory, the easier will be the study of a case, for they are the nucleus around which all the other symptoms group themselves.

3. A man who is suffering and at present emaciated complains of steady pain and pressure on the left side, about the mammillar line, and he had to relinquish his work, as he could not bear any pressure around his waist. The pain becomes aggravated without cause, is worse at night, so that he must get up and walk the floor or he runs out in the street and walks around the church, which is close by and where the posts of the fence allow him some support during a paroxysm of pain. Obstinate constipation without any desire for stool, fullness in the gastric region, worse when eating, nausea after eating, sometimes vomiting, sour taste in mouth, sometimes belching, vertigo. Objective examination reveals nothing, except some sensitiveness to pressure at the painful spot. Lycopodium$^{30}$, three pillules every morning (low potencies of this drug fail to be of any benefit). Constipation, fullness when eating (Nux-vomica has fullness an hour after eating), sour taste (Carbo-veg. has foul, rotten taste) and the belching decided the choice. Gradual improvement follows, so that he took his medicine only once a day and then after a week's severe aggravation. He has to rise again and to walk slowly about in his room ; there is a pressure reaching to the angulus scapulæ. Rhus-tox. worse at night, better by motion, but its pains are around the joints, and cold air aggravates all sufferings. Argentum affects left side, while the splinter-sensation of Agentum-nitricum belongs to the Nitric acid ; it belongs to the carbo-nitrogenous constitution, and has a pain between the fifth and sixth ribs. Magnesia-muriatica, worse at night, better by motion. Ferrum did help in some similar cases, and here the patient complained of dyspnœa and oppression of chest, when he tried to remain in bed in spite of the pain. On account of this tendency to suffocation he received Ferrum-muriaticum$^{3x}$, three times daily three drops, with steady improvement, so that after a few weeks he felt hale and hearty, and every function in his body normal. What pathological condition was the matter with him ? One physician diagnosed ulcus pepticum, another one carcinoma. Dr. L. considered it an obscure abscess, and Koch's lymph-tuburculinum, and each and all failed to give him the least relief. What was the diagnosis ? Dahlke feels unable to give one, but he knew how to cure his patient, and such unscientific treatment is of more value to the patient than to mystify him with empty phrases, and autopsies are of little benefit to the sufferer himself, though a great boon to pathological anatomy.

4. A little child was taken with dyspnœa, alæ nasi symptom, and high fever, whistling râles in chest, dry, hot skin, etc. Aconite seemed to be indicated, but somewhere Trinks has affirmed that Aconite is of very little account in infantile broncho-pneumonia, and following his advice, Belladonna and then Phosphorus$^{5c}$ dec., singly at first and then in alternation, were given, but the child got decidedly worse, and the mucous râles with scanty cough hinted strongly at Antimon-tart.$^{3}$, every three hours a dose. Next day status idem, medicine continued, but after an apparent amelioration a decided aggravation followed, the child lies apathetic, with closed eyes and pale, hot face. With every cough the child turns livid, raises itself up, and then falls back exhausted, covered with sweat. Now in his despair, Dahlke remembered the three forms of pneumonia mentioned by Rademacher, the Nitrum, Ferrum, and Cuprum pneumonia, and finally concluded to try Cuprum-aceticum$^{4}$ dec., three drops every two hours. He dreaded the morning visit, but was pleasantly astonished to find the child greatly improved. It was playful and cheerful, and wanted food. The cough was still spasmodic, but of less intensity and duration, and with the *simillimum* applied the child soon recovered, to the joy of its parents.

[Non in magistro jurare! Trinks may give some good advice, but we all know the beneficial action of Aconite in infantile diseases, especially in the first stage of a disease, be it croup or pneumonia. The doctor gave Phosphorus too early, and it can only be indicated when consolidation of the lung tissue took place. Antimonium-tartar is a two-edged sword, but was a good prescription as the liver hinted at threatening asphyxia. Rademacher and Schüssler compliment one another, and Schüssler's Ferrum-phosphoricum often supplants in our neurasthenic age the use of the sthenic Aconite. In my therapeutics I give clear indications of Ferrum-phos. and of Cuprum, which in the lobular pneumonia of children may become our sheet-anchor.—S. L.]

5. A woman came to the office complaining that for the last six years her menses were irregular, too early and too copious. Two years ago she had an abortus, and now she flows for over a month, the blood dark, in clots, but not of bad odor. Even in the horizontal position she flows steadily, worse at night than in daytime, and aggravated by every motion. Abdominal pains from sacrum forwards; dull headache, vertigo, inappetence, constipation, palpitations, poor sleep. Objectively, retroflexion of the uterus, with position to the right side. Curetting was recommended, but objected to at present. Several years before Ustilago acted splendidly in a similar case and Ustilago was prescribed; a fatal error, for in the latter case the blood was brightened and the flow painless; the flow was in the present case accompanied by pains, the blood dark and clotted; the former case has copious leucorrhœa, the present case none at all, and certainly no improvement could be expected. We meet dark clotted flow in Crocus, Cocculus, China, Chamomilla, Nuxmoschata, etc., but the pain from sacrum forward is specially found under Sabina, which, on the contrary, has a bright red flow. Again, our patient is worse by motion, worse at night than in daytime, a symptom characteristic of Bovista, prescribed 3d dec., every two hours. In the afternoon flooding increased; the drug was changed to Secale, five drops every hour, which relieved her greatly, and after three days the discharge ceased. Flooding worse at night is also found under Magnesia-carbonica and Ammonium-muriaticum and Carbonicum.

[May we be permitted to remind the worthy doctor that Sabina has too early and too profuse menses, too long and debilitating, partly fluid, *partly clotted and offensive*, bright red or dark and clotted, flowing in paroxysms, offensive leucorrhœa, all of them contra-indications to the case. If only Dr. Dahlke could be persuaded to use the higher potencies, or if he only would have allowed Bovista to complete the cure after such a severe aggravation. Why is that glorious tincture of time so much neglected and the *vis medicatrix naturæ* so little credit allowed, when the ball was once set in motion? We still believe Bovista cured the case, for Secale has neither aggravation at night or by motion.—S. L.]

6. A woman has her courses every two weeks, sometimes even every eight days; worse at night; the discharge is sometimes dark, clotted, sometimes light-colored, watery, with abdominal pains and bearing down, especially during defecation, so that she dreads it; great vertigo off and on at any time, worse in the morning when she arises from her bed, none in daytime, even after stooping; no palpitation, no dyspnœa when standing or from quick movements, only excessive lassitude; periodical headache from the nape over vertex reaching the right eye; no nausea or vomiting, but great sensitiveness to noise; cold feet, flushes, foul taste; water-brash, especially mornings; constipation with desire for stool; tongue white in the centre. She looks pale and emaciated, anxious and suffering features, feels unhappy, weeps easily, iritated from small causes and worse from consolation, acrid and corroding leucorrhœa, with itching and burning; the eyes burn when reading and the letters run together. After rejecting many remedies, Dahlke chose Natrum-muriaticum, though it has scanty menses and even amenorrhœa; but the anemia and emaciation, the downheartedness with excessive irritability worse from consolation and the acrid leucorrhœa are symptoms just as characteristic of Natrum-mur. as they contra-indicate Pulsatilla or Sepia. Gradual improvement and cure.

[Hahnemann had already taught that the mental symptoms of the patient take front rank in the solution of the remedy, and when Prof. T. F. Allen teaches that it is of great importance to study out the peculiar symptoms characteristic of the patient, he hits the right point, for we learn more from them than from the symptoms peculiar to the pathological state of the case, and it is this peculiar study of the patient which differentiates the full-fledged physician from the pathological and clinical homœopath. We thank Dr. Dahlke for his extremely interesting cases, and beg him to soar higher than he does now, when he is held down too much by material doses. Try, try again, and the results will be satisfactory.—S. L.]

